# The association between self-esteem and suicidal risk: a meta-analysis

**DOI:** 10.1192/j.eurpsy.2022.2162

**Published:** 2022-09-01

**Authors:** F. Lippo, F. Madeddu, M. Fornaro, R. Calati

**Affiliations:** 1 University of Milan-Bicocca , Department Of Psychology, Milan, Italy; 2 Federico II University of Naples, Department Of Psychiatry, Naples, Italy

**Keywords:** Suicide, self-esteem, suicidal risk, meta-analysis

## Abstract

**Introduction:**

Background: Existing evidence poses low self-esteem as a risk factor for both suicidal ideation (SI) and suicide attempts (SAs) in the general population.

**Objectives:**

The present study assesses the relationship between self-esteem level and SI/SA, considering across the lifespan. Two separate meta-analyses, one for SI and the other for SA are herein reported since they substantially overlap in terms of eligibility procedures and search strategies.

**Methods:**

Eligible studies documented at least one suicidal, and a non-suicidal group. Data were analyzed using the Cochrane Collaboration Review Manager Software (RevMan, version 5.4.1) under the random-effects models. Values were standardized owing to the anticipated heterogeneity of self-esteem rating tools. Sensitivity analyses were performed to control for heterogeneity.

**Results:**

Out of 3,310 initial hits, 24 studies were deemed eligible for inclusion. The meta-analyses showed that individuals with lower levels of self-esteem, compared to those with higher levels, were more likely to endorse both SI and SA. SI reached a standardized mean difference of -0.43 (CI: -0.81, -0.05), while SA reduced by -0.89 (CI: -1.02, -0.76), overall. Limitations: The herein presented results rely on standardized mean differences rather than odds of either SI or SA since the original studies failed to systematically fetch rates of the events.

**Conclusions:**

Lower levels of self-esteem represent a risk factor for both SI and SA across the lifespan. Forthcoming studies should systematically account for multiple moderators to allow meta-analytic synthesis including sub-group and meta-regression analyses assuming high-heterogeneity would still be concerned.

**Disclosure:**

No significant relationships.

